# Supplementary material for: Controlling Floquet states on ultrashort time scales
Source: Nat Commun. 2022 Nov 19;13:7103. doi: 10.1038/s41467-022-34973-4 (PMC9675778; doi:10.1038/s41467-022-34973-4)
Supplement: Supplementary file 1 — Supplementary Information [file 41467_2022_34973_MOESM1_ESM.pdf]

Supplementary information for: Controlling Floquet states on the ultrashort time scale

Matteo Lucchini[1,2*], Fabio Medeghini[1], Yingxuan Wu[1,2], Federico Vismarra[1,2], Rocío Borrego-Varillas[2], Aurora Crego[2] Fabio Frassetto[3], Luca Poletto[3], Shunsuke A. Sato[4,5], Hannes Hübener[5], Umberto De Giovannini[5,6], Angel Rubio[5,7], Mauro Nisoli[1,2]

[1]Department of Physics, Politecnico di Milano, 20133 Milano, Italy
[2]Institute for Photonics and Nanotechnologies, IFN-CNR, 20133 Milano, Italy
[3]Institute for Photonics and Nanotechnologies, IFN-CNR, 35131 Padova, Italy
[4]Center for Computational Sciences, University of Tsukuba, Tsukuba 305-8577, Japan
[5]Max Planck Institute for the Structure and Dynamics of Matter, 22761 Hamburg, Germany
[6]Università degli Studi di Palermo, Dipartimento di Fisica e Chimica, I-90123 Palermo, Italy
[7]Center for Computational Quantum Physics (CCQ), The Flatiron Institute, New York 10010, USA

*To whom correspondence should be addressed; E-mail: matteo.lucchini@polimi.it.

S1 Materials and Methods

In all the experiments performed, the few-femtosecond extreme-ultraviolet (XUV) pulses are generated by focusing 800-μJ infrared (IR) pulses with time duration of about 35-40 fs and central wavelength of 811 nm onto a static gas cell filled with Ar. The 23rd harmonic (H23), centered around 35 eV, is selected by a time-delay compensated monochromator (TDCM) (*1*) while preserving its time duration (*2*). Another portion of the same IR beam (about 1 mJ) is sent to a fiber compression system (*3*), composed by a hollow-core fiber filled with Ne and a set of broad-band chirp mirrors. After compression the IR beam passes through a $\lambda/2$-waveplate

before being reflected by a wide-band polarizer. In this way the pulse duration can be adjusted by changing the Ne pressure in the fiber, while its energy is set by controlling the orientation of the $\lambda/2$-waveplate and thus the fraction of the beam that is reflected by the wide-band polarizer. Before recombining the IR and XUV beams with a drilled mirror, the IR pulses are sent to a delay line controlled by a piezo stage which assures sub-fs precision. Both beams are independently focused onto the target area of a time-of-flight (TOF) spectrometer used to collect the photoelectrons which result by the two-color photoionization of Ne atoms. In the following we address in detail the procedures and data analysis used with quasi-monochromatic pulses for the static measurements, and with short pulses for the pump-probe experiments.

S1.1 Quasi-monochromatic IR pulses

Such pulses are obtained by applying an interferometric filter directly at the IR pulses generated by the laser source, bypassing the fiber-compression setup. The filter has a bandwidth of 10 nm, centered around a wavelength of 800 nm. Temporal characterization with a second-harmonic FROG (*4*) revealed a time duration of about 150 fs (red curve in Fig. 1e of the main manuscript) and negligible spectral chirp. To obtain the data of Fig. 2 of the main manuscript, we first perform a quick pump-probe experiment to find the zero delay between the quasi-monochromatic IR and XUV pulses. Once the temporal overlap has been achieved, the IR pulse energy is varied by changing the angle of the $\lambda/2$-waveplate to control the pulse intensity in the focus. This latter is estimated starting from the IR temporal intensity profile obtained with the FROG, measuring the beam spatial profile at the focus and the power transmitted by the waveplate-polarizer assembly with a powermeter.

Each set of data is acquired by varying the IR intensity in a random order to avoid a systematic error. At a fix intensity, after electrical background removal, the total count of electrons in each SB (i.e. the SB signal) is obtained by integrating the photoelectron spectrum in a 1-eV

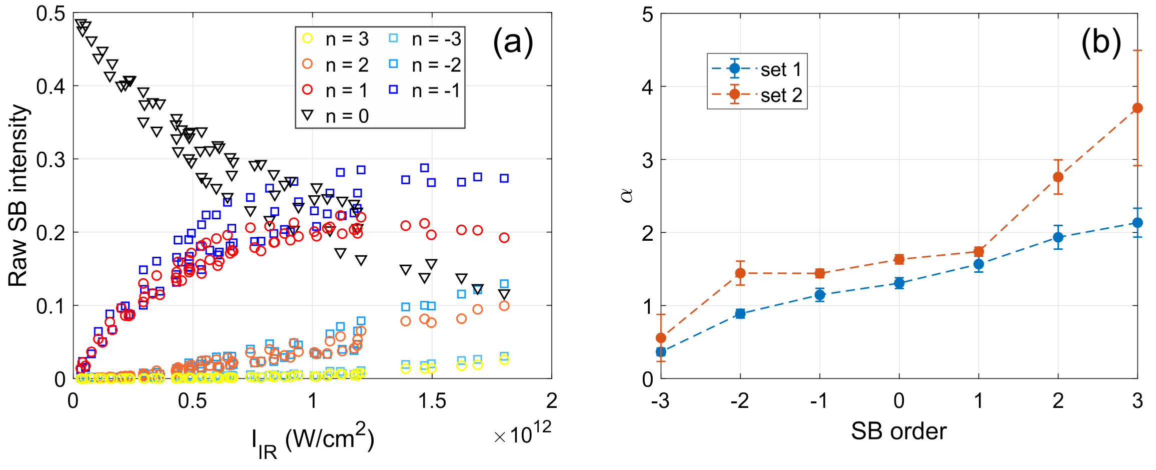

Figure S1: **(a)** Raw SB normalized intensities as extracted from the photoelectron spectra obtained with a quasi-monochromatic IR pulse. **(b)** Absolute electron counts calibration factor for the two different sets of TOF voltages used in the experiment.

energy region around the corresponding SB peak. It is important to notice that since the photoelectron spectra are acquired with a TOF spectrometer, the absolute value for the photoelectron counts is not given. To circumvent this problem we normalized the SB signal by the total area of the XUV-only photoelectron spectrum, I_0. The results reported in Fig. S1(a) show the expected behaviour with I_{IR}, even if the absolute value of the SB normalized intensity is lower than what predicted by the theory. This discrepancy originates from a non-flat energy transfer function of the TOF spectrometer which usually depends on the actual voltages applied to its electrostatic lenses and on the geometry of the interaction. To estimate the non-flat response of the TOF we proceeded as follows. For a chosen SB order n, we considered separately the raw data in Fig. S1(a) which have been taken under the same experimental conditions. Then, using a fitting procedure, we calculated the parameter α_n which minimizes the rms distance between the raw data and the theoretical prediction $A_n^2 = \tilde{J}_n\left(-p_n\frac{E_0}{\omega_0^2}, -\frac{U_p}{2\omega_0}\right)$. The values of α_n as a function of the SB order for the two sets of lenses used in the experiment are reported in Fig. S1(b). The vertical error bars indicate the confidence interval associated with the estimation of α_n. We find

that the calibration factor α_n grows almost linearly with n between 0.5 and 4. Once the calibration factor has been evaluated, the experimental data can be normalized before being directly compared to the theoretical curves (Fig. 2c of the main manuscript). For a given SB, the total relative error associated to the estimation of α_n, represented by the shaded areas in Fig. 2c of the main manuscript, is calculated by summing the squared confidence intervals of the two sets reported in Fig. S1(b).

S1.2 Short IR pulses

To generate short IR pulses of controlled duration, the IR beam is sent to a hollow-core fiber compression setup before passing through the broad-band $\lambda/2$-waveplate and the polarizer. Also in this case, the time duration of the IR pules is measured with the FROG technique (Fig.1e of the main manuscript) while the intensity is set to $\sim 5 \times 10^{11}$ W/cm^2 by adjusting the transmitted power with the $\lambda/2$-waveplate. We performed several pump-probe experiments by collecting the photoelectron spectra while the XUV-IR delay was changed with a step of 3-4 fs (smaller step were used with shorter IR pulses). The resulting spectrograms were mediated to obtain the final spectrogram and reduce the experimental noise. A photoelectron spectrum without the IR was measured each 5 delay steps by blocking the beam with a mechanical shutter during the scan. This XUV-only spectrum is used both to normalize the SB signal as discussed in the main text and to estimate: (i) the variation of the XUV photon flux during the measurement, (ii) the electronic noise which produces a non-zero background below the SB peaks. The raw delay-dependent SB signal was obtained by integrating the spectrogram in energy after the effects (i) and (ii) had been corrected for. The SB intensity is then given by the maximum of the delay-dependent signal which can be estimated, for example, with a Gaussian fit of the energy-integrated delay-dependent SB profile. The raw results are reported in Fig. S2. While each SB signal qualitatively follows the theoretical prediction of Eq. (4) of the main manuscript, we

notice some discrepancies which are related to the not perfect Gaussian shape of the pulses and to the residual variations of the IR central wavelength and intensity from the nominal values. This effects can be accounted for as described in the next session.

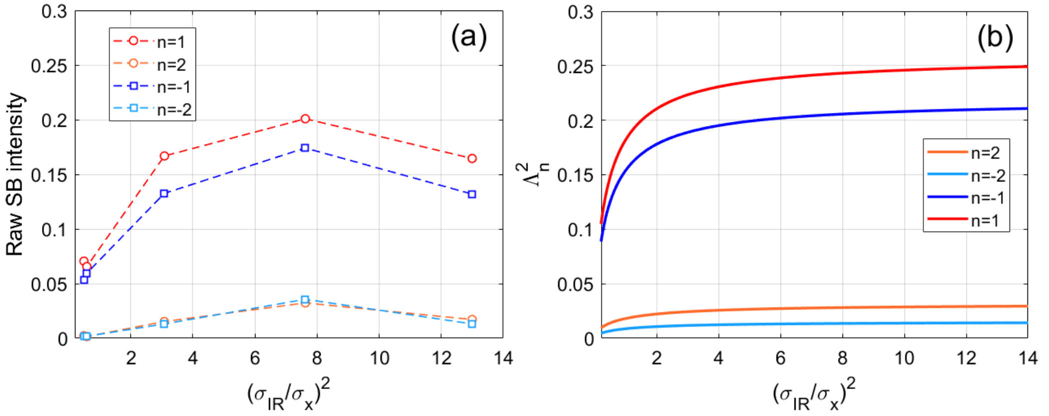

Figure S2: **(a)** Raw SB normalized intensities as extracted from the photoelectron spectrograms obtained with short IR pulses. **(b)** Theoretical prediction for perfect IR Gaussian pulses centered around 800 nm and with $I_{IR} = 5 \times 10^{11}$ W/cm^2.

S1.2.1 Pulse temporal reconstruction

From the shape of Eq. (4) of the main manuscript it appears to be clear that small variations of the IR central wavelength and intensity can influence the value of Λ_n. Therefore, it is crucial to estimate the exact experimental parameters to be able to recast the results over a common nominal IR intensity and wavelength. One possibility is to use ptychographic reconstruction algorithms to extract both the IR and XUV characteristics directly from the experimental spectrogram (*2, 5*). Before applying the reconstruction algorithms one has to characterize the finite spectral resolution of the TOF spectrometer and acquisition system, which may artificially broaden the photoelectron spectrum. To estimate the TOF response, we changed the XUV spectral bandwidth between 200 and 400 meV (full width half maximum, FWHM) by varying the aperture of the central slit of the TDCM, and measured the associated XUV-only photoelec-

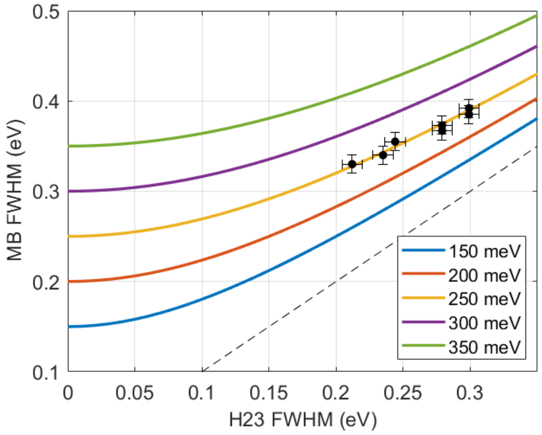

Figure S3: Photoelectron spectral width measured with the TOF spectrometer as a function of the radiation spectral width measured with the XUV spectrometer (black full markers). The solid line represent the expected behavior obtained by convoluting the radiation bandwidth with the TOF energy resolution (reported in the legend).

tron spectra (black markers in Fig. S3). For an ideal spectrometer the two quantities should correspond (black dashed line). In our case we observe that the photoelectron FWHM is generally bigger than the FWHM measured by the XUV spectrometer at the end of the beamline, suggesting that the TOF response can be modeled by a Gaussian bell with a FWHM width of 250 meV (yellow curve in Fig. S3). As the photoelectron spectrum significantly deviates from the XUV spectrum for such harmonic pulses, the application of a standard reconstruction algorithm, which assumes the two quantities to be identical, will strongly underestimate the time duration of the XUV pulse and overestimate the duration of the IR pulse. To avoid this issue, we developed a reconstruction procedure based on the SFA formulation of the photoelectron signal (Eq. (S1)) and capable to account for the actual XUV spectrum and the TOF response function. In brief, following a common approach used in the reconstruction of FROG-like measurements, the photoelectron spectrum is described by the Fourier transform of the product between a pulse (the XUV field) and a pure phase gate (the exponential which depends on the IR). Starting from an educated guess of pulse and gate, the algorithm corrects these quantities while imposing the

amplitude of the simulated spectrogram to be identical to the experimental one. To improve the accuracy, in the present case we forced the code to assume the IR temporal envelope to be the one independently measured with a second-harmonic FROG, the TOF response function to be Gaussian with a width of 250 meV, and the XUV spectral intensity to be identical to the one measured by the XUV spectrometer (assumed to be more reliable as this spectrometer has a resolution $\leq 50\,\mathrm{meV}$ in this range). Moreover, to minimize the effect of the experimental noise, the algorithm directly runs over the differential spectrogram (pump-probe trace minus the XUV-only spectrum). An example of reconstruction is reported in Fig. S4 for the same data of Fig. 3b of the main manuscript. The code provides the XUV spectral phase (Fig. 1b

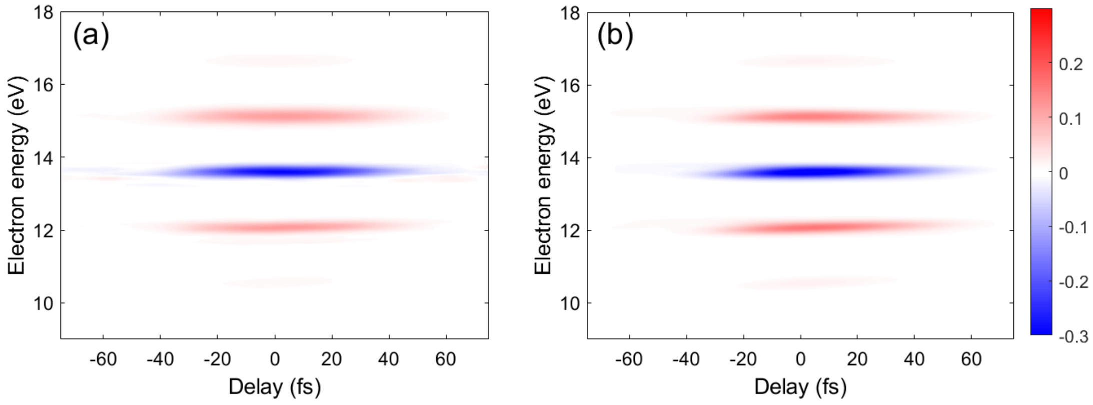

Figure S4: **(a)** Differential photoelectron spectrogram obtained with 45-fs IR pulses. **(b)** Differential spectrogram reconstructed by the fitting procedure.

in the main manuscript), the IR intensity, time duration and wavelength. The parameters extracted for each data point displayed in Fig. 3c of the main manuscript are reported in Fig. S5. As expected the IR wavelength, λ_{IR} (Fig. S5(a)), observes a non-negligible blue shift while moving towards shorter pulses. The IR intensity on target (Fig. S5(b)) ranges between 4.6 and 10.6×10^{12} W/cm^2, while the reconstructed XUV FWHM time duration, σ_x, is constant across the different measurements ($\sigma_x = 12.3 \pm 0.4\,\mathrm{fs}$). In order to set the correct pulse energy, the

IR intensity is estimated in real time before performing the pump-probe scan by measuring the IR temporal and spatial profiles. While the temporal measurement is sufficiently accurate, the FROG setup has been calibrate and tested against the results obtained on target, the measurement of the spatial profile is affected by a considerable background due to the limitations of the detection system. As a results, the IR intensity that is estimated in real time in the lab suffers by a poor accuracy. Furthermore, the effective IR intensity felt by the Ne atoms depends on the degree of spatial overlap at the target position. For these reasons the reconstruction procedure allows us to obtain a more accurate estimation of the effective I_{IR} and correctly calibrate the results. It is important to stress that while both the reconstruction procedure and our model start from the SFA description of the photoelectron spectrogram, they are not based on the same approximations. For example, the reconstruction algorithm applies a global CMA ($p \rightarrow p_c$) instead of considering the SB momenta p_n. The approximations introduced in the reconstruction algorithm have been tested and proved to hold under the experimental conditions used in this work, thus justifying its employment.

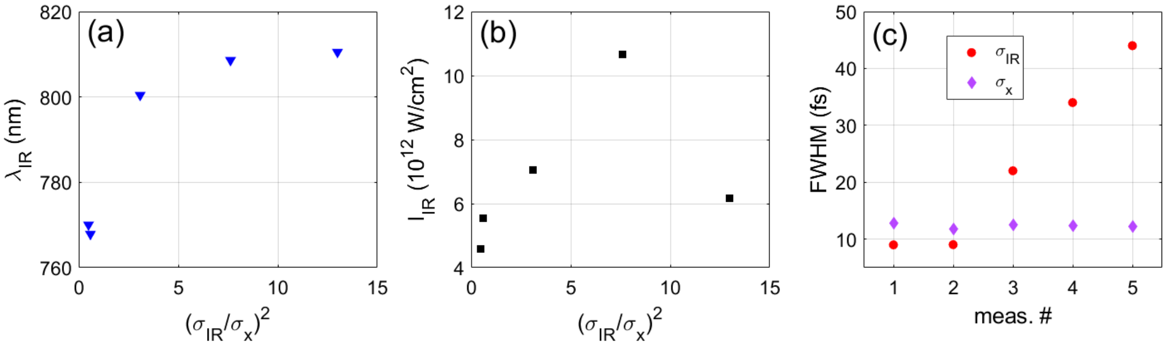

Figure S5: **(a)** Central wavelength of the IR pulses used in the experiment. **(b)** Estimated IR intensity for each spectrogram performed. **(c)** Reconstructed IR (red dots) and XUV (violet diamonds) FWHM pulse durations.

Once the actual pulse parameters are known, it is possible to account for the effect of their deviation from the nominal values, i.e. $I_{IR} = 5 \times 10^{12}$ W/cm^2, $\lambda_{IR} = 800$ nm and perfect

Gaussian envelope. To do so, we compared the values of the SB normalized amplitudes, Λ_n^2, calculated for the actual pulses reconstructed by the fitting procedure (open triangles in Fig. S6(a)), with the values predicted by Eq. (4) of the main manuscript when evaluated for the same time duration of the pulses, but at the nominal values of the other parameters (solid lines in Fig. S6(a)). In this way it is possible to correct the experimental data of Fig. S2(a) (full

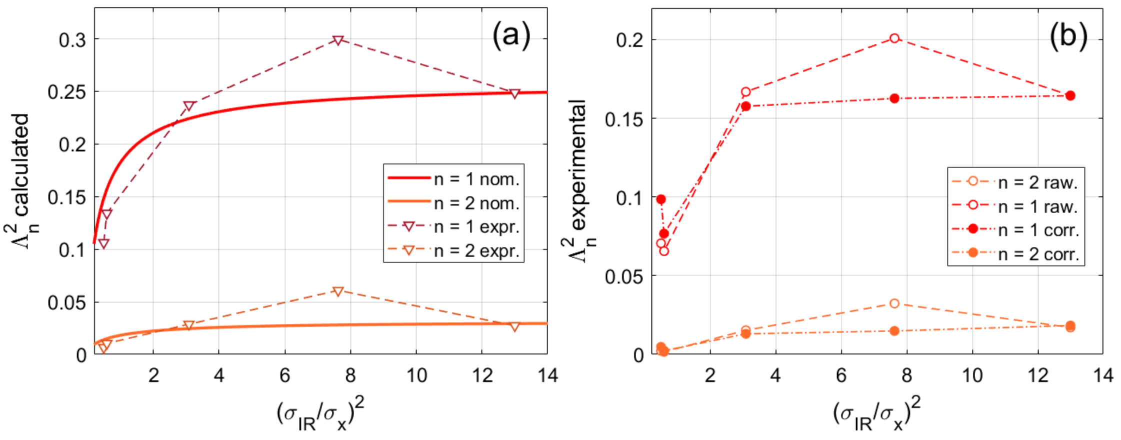

Figure S6: **(a)** SB normalized amplitude Λ_n^2 calculated with the analytical model of Eq. (4) of the main manuscript for ideal Gaussian pulses, $\lambda_{IR} = 800\,\text{nm}$ and $I_{IR} = 5 \times 10^{12}$ W/cm^2, solid curves, or calculated using the actual pulses reconstructed from the experimental spectrograms and described by the parameters in Fig. S5, open triangles. **(b)** Experimental SB normalized amplitudes as extracted from the spectrograms, open circles (same as in Fig. S2(a)), or corrected for the theoretical deviations due to the not nominal IR and XUV parameters, full circles.

circles in Fig. S6(b)) for the theoretical deviation due to the difference between the actual and reference experimental pulse parameters. The result for the positive SBs is displayed by the full circles in Fig. S6(b) and shows an improved agreement with the model of Eq. (4) of the main manuscript. Finally, before comparing the experimental data with the theoretical prediction in Fig. 3b of the main manuscript, the TOF calibration factor α_n is calculated as described for the quasi-monochromatic results (Sec. S1.1) and the associated uncertainty is recast over the theoretical curves (shaded ares in Fig. 3b of the main manuscript).

S2 Theoretical methods

In this section we describe in details the numerical models used for the calculations and simulations. Atomic units are used throughout the document.

S2.1 SFA model

Within the strong-field approximation (SFA), the collection of photoelectron spectra obtained by ionizing an atom of ionization energy I_p with an XUV field $E_x(t)$ and an IR field $E_{IR}(t) = -\frac{dA_{IR}(t)}{dt}$ is (*6*):

$$S(\omega,\tau) = \left| \int_{-\infty}^{\infty} d\,(p + A_{IR}(\,t))E_x(t-\tau)e^{-i\int_t^{\infty}\frac{1}{2}(p+A_{IR}(t'))^2dt'}e^{iI_pt}dt \right|^2, \tag{S1}$$

where we assumed the IR and XUV fields to be linearly polarized with parallel polarization and we considered only the photoelectrons emitted along the polarization direction. We note that in atomic units $\omega = p^2/2$.

If we assume the dipole to be constant in the momentum range under consideration, by exploiting the relation $\int_t^{\infty} dt' = \int_{-\infty}^{\infty} dt' - \int_{-\infty}^{t} dt'$ and given that the integral of $\int_{\infty}^{-\infty} \frac{1}{2}\,(p + A_{IR}(\,t'))^2dt'$ results in a constant phase term which does not affect that final spectrogram of Eq. (S1), we can write:

$$S(\omega,\tau) = \left| \int_{-\infty}^{\infty} E_x(t-\tau)e^{i\int_{-\infty}^{t}[pA(t')+\frac{1}{2}A(t')^2]dt'}e^{i\left(I_p+\frac{p^2}{2}\right)t}dt \right|^2, \tag{S2}$$

All the simulations performed in this work are obtained by numerical evaluation of Eq. (S1) and assuming the IR and XUV pulses to be Gaussians of the form:

$$E_{IR}(t) = E_0(t)\sin(\omega_0 t) = E_0 e^{-\frac{t^2}{\gamma_r^2}}\sin(\omega_0 t) \tag{S3}$$

$$E_X(t) = E_{x0}(t)(e^{-i\omega_x t} + cc.) = E_{x0}e^{-\frac{t^2}{\gamma_x^2}}(e^{-i\omega_x t} + cc.), \tag{S4}$$

where the complex quantities $\gamma_x = 2\sqrt{\left(\frac{c}{2}\right)^2 - i\frac{\beta_x}{2}}$ and $\gamma_r = 2\sqrt{\left(\frac{a}{2}\right)^2 - i\frac{\beta_r}{2}}$ depend on the group-delay dispersion (GDD) coefficients β_x and β_r, and the XUV and IR intensity FWHM

time durations, $\sigma_x = c\sqrt{2\log(2)}$ and $\sigma_r = a\sqrt{2\log(2)}$.

S2.2 Floquet (Volkov) states

Let's consider a free electron described by the following plane wave:

$$\psi_0(\mathbf{r}, t) = \frac{1}{(2\pi)^{3/2}} \exp\left\{ i \left[\mathbf{p} \cdot \mathbf{r} - \frac{1}{2} p^2 t \right] \right\} \tag{S5}$$

In the presence of an additional *monochromatic* IR field,

$$A_{IR}(t) = \frac{E_0}{\omega_0} \cos(\omega_0 t) \tag{S6}$$

the wave function of the free electron is described by the Volkov wave (*7*):

$$\psi_V(\mathbf{r}, t) = \frac{1}{(2\pi)^{3/2}} \exp\left\{ i \left[\mathbf{p} \cdot \mathbf{r} - \int_0^t dt' \frac{1}{2} \left(\mathbf{p} + \mathbf{A}_{IR}(t')\right)^2 \right] \right\} \tag{S7}$$

where $\mathbf{p}$ is the electron momentum and $\mathbf{r}$ is the position vector. Following what reported by Madsen (*8*), and assume that we collect electrons whose momentum $\mathbf{p}$ is parallel to the polarization of the IR pulse and XUV pulses, the Volkov wave can be rewritten as:

$$\psi_V(\mathbf{r}, t) = \frac{1}{(2\pi)^{3/2}} e^{i\mathbf{p}\cdot\mathbf{r}} \sum_{n=-\infty}^{\infty} \tilde{J}_n \left(-p\frac{E_0}{\omega_0^2}, -\frac{U_p}{2\omega_0} \right) e^{-i\left(p^2/2 + U_p - n\omega_0\right)t}. \tag{S8}$$

where $\tilde{J}_n$ indicates the generalized Bessel function defined as the following sum of products of ordinary Bessel functions:

$$\tilde{J}_n(x, y) = \sum_{j=-\infty}^{\infty} J_{n-2j}(x) J_j(y). \tag{S9}$$

If we neglect the spatial dependence, the time-dependent part of the Volkov state can be rewritten in the form:

$$\psi(t) = e^{-i\varepsilon t} \sum_{n=-\infty}^{\infty} A_n e^{in\omega_0 t}, \tag{S10}$$

where

$$\begin{cases} \epsilon = p^2/2 + U_p \\ A_n = \tilde{J}_n\left(-p\frac{E_0}{\omega_0^2}, -\frac{U_p}{2\omega_0}\right). \end{cases} \tag{S11}$$

Using the dipole approximation, the photoelectron spectrum resulting from the ionization of an atomic state $|0\rangle = e^{iI_pt}\phi_0(\mathbf{r})$ with a field $E_x(t)$ into a Volkov state $\psi_V(\mathbf{r},t)$ is then given by (*8*):

$$\left|\int_{-\infty}^{\infty} dt\langle\psi_V(t)|\mathbf{A}_x(t)\cdot\hat{\mathbf{p}}|0\rangle\right|^2 \simeq \left|\int_{-\infty}^{\infty}\psi_V^*(t)\mu\, E_x(t)\phi_0(\mathbf{r})e^{iI_pt}dt\right|^2. \tag{S12}$$

If the Fourier transform of the spatial part of the initial atomic state $\phi_0(\mathbf{r})$ does not vary significantly in the energy region of interest, i.e. if the atomic dipole $d_n = \langle\frac{e^{i\mathbf{p}\cdot\mathbf{r}}}{(2\pi)^{3/2}}|\mu|\phi_0(\mathbf{r})\rangle \simeq 1$, we get:

$$\left|\int_{-\infty}^{\infty} dt\langle\psi_V(t)|d\,E_x(t)|0\rangle\right|^2 \simeq \left|\int_{-\infty}^{\infty} E_x(t)\sum_{n=-\infty}^{\infty}\tilde{J}_n\left(-p\frac{E_0}{\omega_0^2}, -\frac{U_p}{2\omega_0}\right)e^{i\left(p^2/2+U_p-n\omega_0\right)t}e^{iI_pt}dt\right|^2, \tag{S13}$$

which yields the same result of the SFA formula, as we will discuss in the next Section.

S2.3 Link between the two models

Starting from Eq. (S2) and assuming a monochromatic IR field of the form of Eq. (S6), we can developed integral in the phase term into two contributions of the form:

$$i\int_{-\infty}^{t} pA_{IR}(t')dt' = ip\frac{E_0}{\omega_0}\int_{-\infty}^{t}\cos(\omega_0t')dt' = ip\frac{E_0}{\omega_0^2}\sin(\omega_0t), \tag{S14}$$

and

$$i\int_{-\infty}^{t}\frac{1}{2}A_{IR}(t')^2dt' = i\frac{E_0^2}{2\omega_0^2}\int_{-\infty}^{t}\cos^2(\omega_0t')dt' = iU_pt + i\frac{U_p}{2\omega_0}\sin(2\omega_0t), \tag{S15}$$

where we introduced the ponderomotive energy $U_p = E_0^2/(4\omega_0^2)$. We can thus rewrite Eq. (S2) as:

$$S(\omega) = \left|\int_{-\infty}^{\infty} E_x(t)e^{ip\frac{E_0}{\omega_0^2}\sin(\omega_0t)}e^{i\frac{U_p}{2\omega_0}\sin(2\omega_0t)}e^{i\left(U_p+I_p+\frac{p^2}{2}\right)t}dt\right|^2. \tag{S16}$$

Using the Jacobi-Anger expansion, $e^{iz\sin\theta} = \sum_{n=-\infty}^{\infty} J_n(z)e^{in\theta}$, for the two sinusoidal phase terms in the above integral we get (*8*):

$$e^{i(x\sin\theta+y\sin 2\theta)} = \sum_{n=-\infty}^{\infty} \tilde{J}_n(x,y)e^{in\theta}. \tag{S17}$$

where $\tilde{J}_n(x,y)$ indicates the generalized Bessel function of order n defined by Eq. (S9). It is now possible to rewrite Eq. (S16) as:

$$S(\omega) = \left| \int_{-\infty}^{\infty} E_x(t) \sum_{k=-\infty}^{\infty} \tilde{J}_k\left(p\frac{E_0}{\omega_0^2}, \frac{U_p}{2\omega_0}\right) e^{ik\omega_0 t} e^{i\left(U_p+I_p+\frac{p^2}{2}\right)t} dt \right|^2. \tag{S18}$$

Since $-\sin(\theta) = \sin(-\theta)$, starting from the definition of Eq. (S17), it is easy to show that

$$\tilde{J}_n(-x,-y) = \tilde{J}_{-n}(x,y). \tag{S19}$$

Therefore, if we substitute $k = -n$, Eq. (S18) becomes

$$S(\omega) = \left| \int_{-\infty}^{\infty} E_x(t) \sum_{n=-\infty}^{\infty} \tilde{J}_n\left(-p\frac{E_0}{\omega_0^2}, -\frac{U_p}{2\omega_0}\right) e^{-in\omega_0 t} e^{i\left(U_p+I_p+\frac{p^2}{2}\right)t} dt \right|^2, \tag{S20}$$

which proves that the SFA approach and Eq. (S13) yield the same result:

$$S(\omega) = \left| \int_{-\infty}^{\infty} E_x(t) \sum_{n=-\infty}^{\infty} A_n(p, E_0, \omega_0) e^{-in\omega_0 t} e^{i(\varepsilon+I_p)t} dt \right|^2, \tag{S21}$$

with $A_n(p, E_0, \omega_0)$ defined by Eq. (S11).

S2.4 Photoelectron SB signal

If we concentrate on the positive part of the spectrum, use the XUV field definition of Eq. (S4) and consider that in atomic units $p^2/2 = \omega$, we can rewrite Eq. (S21) as:

$$S(\omega) = \left| \int_{-\infty}^{\infty} \sum_{n=-\infty}^{\infty} E_{x0}(t) A_n(p, E_0, \omega_0) e^{i(\omega-\omega_n)t} dt \right|^2, \tag{S22}$$

where we have introduced the SB energy $\omega_n = \omega_x + n\omega_0 - U_p - I_p$. If the XUV pulse bandwidth is narrow enough, the square modulus of the sum in the above equation corresponds to the sum

of the square moduli. Furthermore, within each SB the momentum dependence of A_n can be neglected by substituting $p \to p_n = \sqrt{2\omega_n}$. Therefore, the photoelectron spectrum is given by $S(\omega) = \sum_{n=-\infty}^{\infty} SB_n(\omega)$ where the quantity $SB_n(\omega)$ is the modulus square of the Fourier transform of the XUV field envelope, multiplied by the Floquet ladder amplitude evaluated at the SB central momentum:

$$SB_n(\omega) = \left| A_n(p_n, E_0, \omega_0) \mathcal{F}\left\{ E_{x0}(t) e^{-i\omega_n t} \right\} \right|^2 . \tag{S23}$$

This quantity represents the n-th SB signal which depends, for monochromatic IR fields, only on the final photoelectron energy ω.

If the XUV bandwidth is narrow enough, from Eq. (S22) we get:

$$S(\omega) = \left| \sum_{n=-\infty}^{\infty} A_n(p_n, E_0, \omega_0) \int_{-\infty}^{\infty} E_{x0}(t) e^{i(\omega-\omega_n)t} dt \right|^2 = \tag{S24}$$

$$= \left| \sum_{n=-\infty}^{\infty} A_n(p_n, E_0, \omega_0) \hat{E}_{x0}(\omega - \omega_n) \right|^2 . \tag{S25}$$

where $\hat{E}_{x0}(\omega - \omega_n) = E_{0x} \delta(\omega - \omega_n)$ is the Fourier transform of the XUV envelope shifted in ω_n. In this case the time evolution of the photoelectron wavepacket originating solely from the IR dressing, $s(t)$, can be estimated as:

$$s(t) = F^{-1}\{\sqrt{S(\omega)}\}/E_{0x}(t) = \tag{S26}$$

$$= \sum_{n=-\infty}^{\infty} A_n(p_n, E_0, \omega_0) F^{-1}\{\hat{E}_{x0}(\omega - \omega_n)\}/E_{0x}(t) = \tag{S27}$$

$$= e^{i(\omega_x - U_p - I_p)t} \sum_{n=-\infty}^{\infty} A_n(p_n, E_0, \omega_0) e^{in\omega_0 t}, \tag{S28}$$

and therefore

$$|s(t)|^2 \propto \left| \sum_{n=-\infty}^{\infty} A_n(p_n, E_0, \omega_0) e^{in\omega_0 t} \right|^2 \tag{S29}$$

which represents the time evolution of the scattering amplitude without considering the XUV temporal properties.

S2.5 Driving pulses of finite duration

In case of IR pulses of finite duration the vector potential is given by:

$$A_{IR}(t) = A_0(t)\cos(\omega_0 t), \tag{S30}$$

and the associated electric field is:

$$E_{IR}(t) = A_0(t)\,\omega_0 \sin(\omega_0 t) - \frac{dA_0(t)}{dt}\cos(\omega_0 t) \tag{S31}$$

If the slowly varying envelope approximation (SVEA) can be applied, i.e. if the pulse duration is bigger than its oscillation period $\sigma_{IR} \gg 2\pi/\omega_0$, and $dA_0(t)/dt \sim 0$, then

$$E_{IR}(t) \simeq A_0(t)\,\omega_0 \sin(\omega_0 t) = E_0(t)\sin(\omega_0 t), \tag{S32}$$

where $E_0(t) = A_0(t)\,\omega_0$.

Referring to Eq. (S2), using the fact that the pulse is finite ($A_0(-\infty) = 0$) and under the SVEA ($dA_0(t)/dt \sim 0$), the integral in the IR phase term yields the following two contributions:

$$p\int_{-\infty}^{t} A(t')dt' \simeq \frac{pA_0(t)}{\omega_0}\sin(\omega_0 t) = \frac{pE_0(t)}{\omega_0^2}\sin(\omega_0 t), \tag{S33}$$

and

$$\int_{-\infty}^{t} \frac{A^2(t')}{2}dt' \simeq U_p(t)t + \frac{U_p(t)}{2\omega_0}\sin(2\omega_0 t'), \tag{S34}$$

where we have introduced an "instantaneous" ponderomotive energy of the form: $U_p(t) = E_0^2(t)/(4\omega_0^2)$. The integral in Eq. (S2) can now be rewritten as:

$$S(\omega,\tau) \simeq \left| \int_{-\infty}^{\infty} E_x(t-\tau) e^{i\frac{pE_0(t)}{\omega_0^2}\sin(\omega_0 t)} e^{i\frac{U_p(t)}{2\omega_0}\sin(2\omega_0 t)} e^{i\left(U_p(t)+I_p+p^2/2\right)t} dt \right|^2. \tag{S35}$$

If the pulse envelope $E_0(t)$ evolves on a slower time scale than the carrier period $T_{IR} = 2\pi/\omega_0$, following the two-time approach used in Ref. (*9*) (see Sec. S2.9), it is possible to show that:

$$e^{i\frac{pE_0(t)}{\omega_0^2}\sin(\omega_0 t)} = \sum_{k=-\infty}^{\infty} J_k\left(\frac{pE_0(t)}{\omega_0^2}\right) e^{ik(\omega_0 t)}. \tag{S36}$$

If we extend this approach to the second complex exponential and use the generalized Bessel functions, the spectrogram becomes:

$$S(\omega,\tau) \simeq \left| \int_{-\infty}^{\infty} E_x(t-\tau) \sum_{n=-\infty}^{\infty} \tilde{J}_n \left(-\frac{pE_0(t)}{\omega_0^2}, -\frac{U_p(t)}{2\omega_0} \right) e^{i\left(U_p(t)+I_p+p^2/2-n\omega_0\right)t} dt \right|^2, \quad \text{(S37)}$$

which is identical to Eq. (S20) besides the time dependence in the argument of the generalized Bessel functions. Focusing only on the positive spectrum and expressing the XUV field as: $E_x(t) = E_{x0}(t)e^{-i\omega_x t}$ we can write:

$$S(\omega,\tau) \simeq \left| \int_{-\infty}^{\infty} E_{x0}(t-\tau) \sum_{n=-\infty}^{\infty} \tilde{J}_n \left(-\frac{pE_0(t)}{\omega_0^2}, -\frac{U_p(t)}{2\omega_0} \right) e^{i(\omega-\omega_n)t} dt \right|^2, \quad \text{(S38)}$$

where we omitted the pure phase term $e^{i\omega_x\tau}$ as it disappears with the $|\cdot|^2$. Also in this case, if the XUV bandwidth is small when compared to the IR photon energy, the total spectrum can be seen as the sum of spectrally separated contributions:

$$S(\omega,\tau) \simeq \left| \sum_{n=-\infty}^{\infty} f_n(\omega,\tau) \right|^2 \simeq \sum_{n=-\infty}^{\infty} |f_n(\omega,\tau)|^2 = \sum_{n=-\infty}^{\infty} SB_n(\omega,\tau). \quad \text{(S39)}$$

After applying the central momentum approximation, the SB signal is hence given by:

$$SB_n(\omega,\tau) = |\mathcal{F}\{\, E_{x0}(t-\tau)\tilde{J}_n \left(-\frac{p_n E_0(t)}{\omega_0^2}, -\frac{U_p(t)}{2\omega_0} \right) \}|^2, \quad \text{(S40)}$$

where the symbol $\mathcal{F}$ denotes the Fourier transform.

S2.6 Low-intensity limit

Let's analyze the behavior of the generalized Bessel functions $\tilde{J}_n(x,y)$, for both arguments that tend towards zero. For a standard Bessel function of the first kind it holds:

$$\lim_{x\to 0} J_k(x) = \frac{(\mathrm{sg}(k))^k}{|k|!} \left(\frac{x}{2}\right)^{|k|} \quad \text{(S41)}$$

where $\mathrm{sg}(x)$ donetes the sign of x. We can use the approximation of Eq. (S41) to obtain the following limit for $\tilde{J}_n(x,y)$:

$$\lim_{x,y\to 0} \tilde{J}_n(x,y) = \lim_{x\to 0} \sum_{k=-\infty}^{\infty} J_{n-2k}(x) J_k(y) \tag{S42}$$

$$= \sum_{k=-\infty}^{\infty} \frac{(\mathrm{sg}(n-2k))^{n-2k}}{|n-2k|!} \left(\frac{x}{2}\right)^{|n-2k|} \frac{(\mathrm{sg}(k))^k}{|k|!} \left(\frac{y}{2}\right)^{|k|} \tag{S43}$$

If the field amplitude and the electron final momentum are not too big, than $\frac{U_p(t)}{2\omega_0} \ll \frac{pE_0(t)}{\omega_0^2}$, and for $\frac{pE_0(t)}{\omega_0^2} \to 0$ also $\frac{U_p(t)}{2\omega_0} \to 0$, leading to:

$$\tilde{J}_n\left(-\frac{pE_0(t)}{\omega_0^2}, -\frac{U_p(t)}{2\omega_0}\right) \simeq \sum_{k=-\infty}^{\infty} \frac{(\mathrm{sg}(n-2k))^{n-2k}}{|n-2k|!} \frac{(\mathrm{sg}(k))^k}{|k|!} \times \frac{(-1)^{|n-2k|+|k|}}{2^{|n-2k|+4|k|}} \frac{p^{|n-2k|}}{\omega_0^{2|n-2k|+3|k|}} E_0(t)^{|n-2k|+2|k|} \tag{S44}$$

The weight of the terms in the above equation decreases rapidly with $|k|$, and the terms which give the major contributions are the ones for which $0 < k < n/2$ for $n > 0$ and with $n/2 < k < 0$ for $n < 0$. If $n \neq 0$, the time evolution of those major terms follows $E_0(t)^{|n-2k|+2|k|} = E_0(t)^{|n|} = E_0^{|n|} g(t)^{|n|}$, where we have expressed the pulse envelope $E_0(t)$ as the product of a normalized function $g(t)$ and an amplitude E_0. In view of this limit, we can rewrite $\tilde{J}_n$ as follows:

$$\tilde{J}_n \simeq g(t)^{|n|} \sum_{k=-\infty}^{\infty} \frac{(\mathrm{sg}(n-2k))^{n-2k}}{|n-2k|!} \frac{(\mathrm{sg}(k))^k}{|k|!} \frac{(-1)^{|n-2k|+|k|}}{2^{|n-2k|+4|k|}} \frac{p^{|n-2k|}}{\omega_0^{2|n-2k|+3|k|}} E_0^{|n-2k|+2|k|} \tag{S45}$$

$$\simeq g(t)^{|n|} \tilde{J}_n\left(-\frac{pE_0}{\omega_0^2}, -\frac{U_p}{2\omega_0}\right) \tag{S46}$$

where $U_p = E_0^2/(4\omega_0^2)$. In this limit the temporal evolution of the n-th generalized Bessel follows the n-th power of the pulse normalized envelope times the amplitude of the n-th state of the Floquet ladder (Eq. (S11)) for a monochromatic pulse of amplitude $E_0 = E_0(t=0)$ (corresponding to the pulse maximum amplitude).

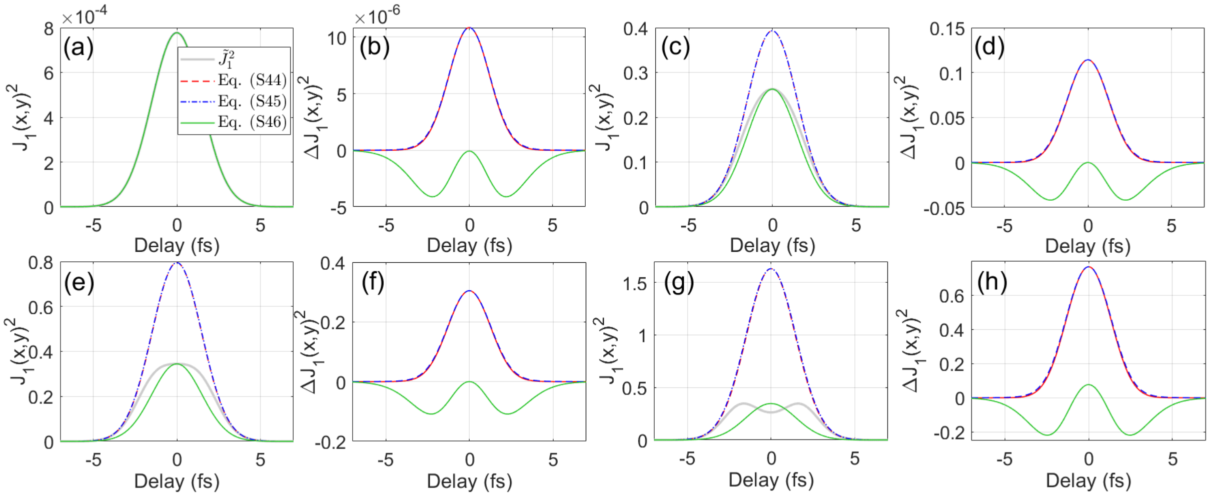

Figure S7: **(a)** Square of the first order generalized Bessel function calculated with the different approximations discussed in the text for a field average intensity of 1×10^9 W/cm^2. **(b)** Difference between the approximated formulas used in (a) and the exact computation. **(c)**, **(d)**, same as (a) and (b) but for an average intensity of 5×10^{11} W/cm^2. **(e)**, **(f)** and **(g)**, **(h)**, refer instead to a field average intensity of 1×10^{12} W/cm^2 and 2×10^{12} W/cm^2, respectively.

For $n = 0$ the generalized Bessel can instead be approximated with:

$$\tilde{J}_0 \simeq 1 - \left(1 - \tilde{J}_0\left(-\frac{pE_0}{\omega_0^2}, -\frac{U_p}{2\omega_0}\right)\right) g(t)^2 = A_0'(t) \tag{S47}$$

While Eq. (S45) has been derived for $pE_0(t)/\omega_0^2 \to 0$, we found Eq. (S46) to be a good approximation on a broader range, i.e. until the Bessel functions in Eq. (S42) are monotonically increasing in their arguments. Figure S7 shows the square of the first order generalized Bessel function $\tilde{J}_1\left(-\frac{pE_0(t)}{\omega_0^2}, -\frac{U_p(t)}{2\omega_0}\right)^2$, calculated for ionization of Neon with H23, an 800-nm IR field with 5-fs FWHM pulse duration, and different field intensities $I_{IR} = \frac{1}{2}\varepsilon_0 c E_0^2$. As it is possible to notice, Eq. (S44) and Eq. (S45) give almost identical results which nicely predict the temporal behavior of $\tilde{J}_1$ at low intensities (below 10^{10} W/cm^2, Figs. S7(a), (b)). The error introduced by the approximation of Eq. (S46) is comparable or smaller. This latter approximation is reasonable up to $I_{IR} = 1 \times 10^{12}$ W/cm^2 (Figs. S7(c)-(f)). Above, the Bessels composing $\tilde{J}_1$ are not all monotonic with their arguments and the function maximum is no longer at $t = 0$.

Therefore the approximation gets worse (Figs. S7(g), (h)).

It is important to underline that if the approximation holds for $\tilde{J}_1$, all the more reasons it will hold for the other orders. Indeed, with increasing order the maximum of the generalized Bessel function is reached later in intensity (Fig. S8(a)). For $n = -1$, instead, the central SB momentum $p{-}1$ is lower (corresponding to the 22nd harmonic instead of the 24th). Figures S8(b), (c) show the square of $\tilde{J}_1$ as a function of time I_{IR}, calculated for the same pulse as in Fig S7 with the exact formula (Fig. S8b) and with the approximated expression of Eq. (S46) (Fig. S8c). Figure S8(d) displays their difference. As it is possible to notice, up to a field average intensity of 1×10^{12} W/cm^2 the approximation of Eq. (S46) is reasonable.

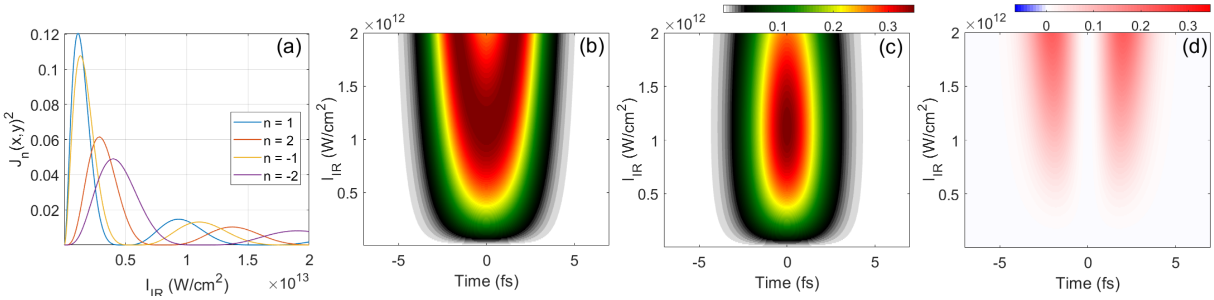

Figure S8: **(a)** Square of the generalized Bessel function calculated for different orders. The electron momentum p is properly adjusted to the Bessel order in the calculations. **(b)** $\tilde{J}_1^2$ calculated with the exact formula or, **(c)**, with the approximation of Eq. (S46). **(d)** Point-by-point difference between (b) and (c).

In the range where the approximation is valid, Eq. (S38) thus becomes:

$$S(\omega,\tau) \simeq \left| \int_{-\infty}^{\infty} E_{x0}(t-\tau) \Big[\sum_{\substack{n=-\infty \\ n\neq 0}}^{\infty} \tilde{J}_n\left(-\frac{pE_0}{\omega_0^2}, -\frac{U_p}{2\omega_0}\right) g(t)^{|n|} e^{i(\omega-\omega_n)t} + \right.$$
$$\left. +1 - \left(1 - \tilde{J}_0\left(-\frac{pE_0}{\omega_0^2}, -\frac{U_p}{2\omega_0}\right)\right) g(t)^2 e^{i(\omega-\omega_0)t} \Big] dt \right|^2, \tag{S48}$$

If the XUV bandwidth is small compared to the IR photon energy then the SB signal ($n \neq 0$) of

Eq. (S40) is now given by:

$$SB_n(\omega,\tau) = \left| \tilde{J}_n \left(-\frac{p_n E_0}{\omega_0^2}, -\frac{U_p}{2\omega_0} \right) \mathcal{F}\left\{ E_{x0}(t-\tau) g(t)^{|n|} \right\} \right|^2 , \tag{S49}$$

which is the same as Eq. (3) of the main manuscript.

In this limit, the time evolution of the scattering amplitude without considering the XUV temporal properties goes as:

$$|s(t)|^2 \propto \left| \sum_{\substack{n=-\infty \\ n\neq 0}}^{\infty} A_n(p_n, E_0, \omega_0) g(t)^{|n|} e^{in\omega_0 t} + A_0'(t) \right|^2 \tag{S50}$$

where $A_0'(t)$ is given by Eq. (S47). Figure S9(a) shows the behavior of $|s(t)|^2$ calculated for a Floquet state (light grey, Eq. (S29)) and for driving pulses of different duration σ_{IR} (Eq. (S50)): 146 fs, dashed-blue, 43.3 fs ciano, 21.1 fs green and 9.2 fs red. The IR intensity used is 5×10^{11} W/cm^2. Hence the curves coincide with the second inset of Fig. 2c, grey curve, and the four insets of Fig. 3c, coloured curves. Figure S9(b) shows the difference between $|s(t)|^2$ of a Floquet state and the that induced of a pulsed driving (same color coding as in Fig. S9(a)), visually representing how Eq. (S50) deviates from the Floquet behaviour of Eq. (S29).

S2.7 Gaussian pulses

If both pulses have a Gaussian envelope of the form of Eqs. (S3) and (S4), the SB signal of Eq. (S49) becomes:

$$SB_n(\omega,\tau) = \left| E_{x0} \tilde{J}_n \left(-\frac{p_n E_0}{\omega_0^2}, -\frac{U_p}{2\omega_0} \right) \int_{-\infty}^{\infty} e^{-\frac{(t-\tau)^2}{\gamma_x^2}} e^{-\frac{t^2}{\gamma_{r,n}^2}} e^{i(\omega-\omega_n)t} dt \right|^2 , \tag{S51}$$

where $\gamma_{r,n} = \gamma_r/\sqrt{|n|}$. The sideband signal in the experimental spectrogram is given by $|S(\omega,\tau)_n|^2$ and it is thus related to the square modulus of the Fourier transform of the product between the two Gaussian bells, $G(t,\tau) = e^{-\frac{(t-\tau)^2}{\gamma_x^2}} e^{-\frac{t^2}{\gamma_{r,n}^2}}$. It is possible to show that $G(t,\tau)$ is another Gaussian function of the form:

$$G(t,\tau) = e^{-\frac{\tau^2}{\gamma_{r,n}^2+\gamma_x^2}} e^{-\frac{(t-\mu_{xr})^2}{\gamma_{xr}^2}} = G'(t-\mu_{xr},\tau) \tag{S52}$$

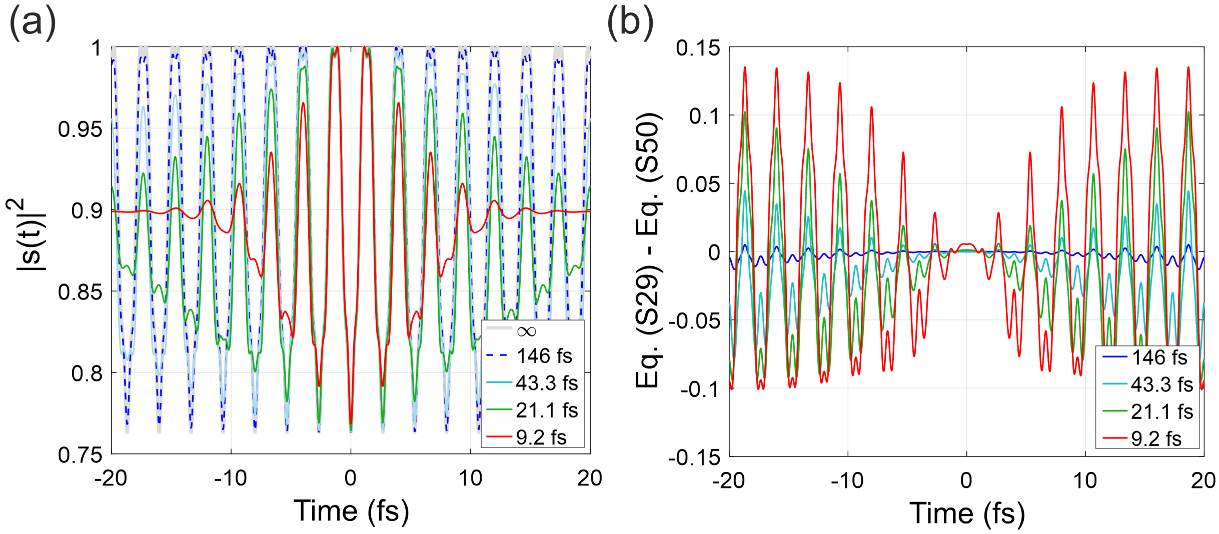

Figure S9: **(a)**, time evolution of scattering amplitude related to the IR pump only, $|s(t)|^2$, calculated for an IR intensity of 5×10^{11} W/cm^2. The grey curve represents the monochromatic case (Floquet state, Eq. (S29)), while the colored curves show the prediction of Eq. (S50) for σ_{IR} equal: 146 fs, dashed-blue, 43.3 fs ciano, 21.1 fs green and 9.2 fs red. These conditions correspond to the experimental points in Figs. 2c and 3c of the main manuscript. **(b)**, Deviations of the pulsed case from the monochromatic scenario. Same color coding as in (a).

where

$$\gamma_{xr} = \sqrt{\frac{\gamma_{r,n}^2 \gamma_x^2}{\gamma_{r,n}^2 + \gamma_x^2}} \tag{S53}$$

$$\mu_{xr} = \frac{\tau \gamma_{r,n}^2}{\gamma_{r,n}^2 + \gamma_x^2} \tag{S54}$$

The Fourier transform in Eq. (S51) can now be solved to obtain (*10*):

$$SB_n(\omega, \tau) = \pi E_{x0}^2 \tilde{J}_n \left(-\frac{p_n E_0}{\omega_0^2}, -\frac{U_p}{2\omega_0} \right)^2 \frac{\gamma_{r,n}^2 \gamma_x^2}{\gamma_{r,n}^2 + \gamma_x^2} e^{-\frac{2\tau^2}{\gamma_{r,n}^2 + \gamma_x^2}} e^{-\frac{\gamma_{xr}}{2}(\omega - \omega_n)^2}, \tag{S55}$$

which integrated in energy yields:

$$SB_{\omega,n}(\tau) = \int_{-\infty}^{\infty} SB_n(\omega, \tau) d\omega = \pi E_{x0}^2 \tilde{J}_n \left(-\frac{p_n E_0}{\omega_0^2}, -\frac{U_p}{2\omega_0} \right)^2 \frac{\sqrt{2\pi}\gamma_x}{\sqrt{\frac{\gamma_x^2}{\gamma_{r,n}^2} + 1}} e^{-\frac{2\tau^2}{\gamma_{r,n}^2 + \gamma_x^2}}. \tag{S56}$$

If we now evaluate its maximum (i.e. compute the value at $\tau = 0$), and normalize the result by the area of the XUV-only photoelectron spectrum,

$$I_0 = \int_{-\infty}^{\infty} |\hat{E}_x(\omega)|^2 d\omega = \int_{-\infty}^{\infty} E_{x0}^2 \pi \gamma_x^2 e^{-\frac{\gamma_x^2}{2}(\omega-\omega_x)^2} d\omega = E_{x0}^2 \pi \sqrt{2\pi}\gamma_x, \tag{S57}$$

we find the expression of Eq. (4) of the main manuscript:

$$\Lambda_n^2 = \frac{SB_{\omega,n}(0)}{I_0} = \tilde{J}_n\left(-\frac{p_n E_0}{\omega_0^2}, -\frac{U_p}{2\omega_0}\right)^2 \frac{1}{\sqrt{\frac{|n|\gamma_x^2}{\gamma_r^2}+1}} = \frac{A_n^2}{\sqrt{\frac{|n|\sigma_x^2}{\sigma_r^2}+1}}. \tag{S58}$$

This proves that it is possible to extract the Floquet ladder amplitude A_n (Eq. (S11)) directly from the measurements if we correct the normalized SB intensity for the XUV and IR time durations. The higher the SB order, i.e. the higher n in Eq (S58), the stronger the amplitude reduction. Therefore high-order ladder states are more affected by the finite time duration of the IR pulse. As expected, at the long-pulse limit we get:

$$\lim_{\Delta\sigma_r \to \infty} \Lambda_n^2 = A_n^2 \tag{S59}$$

and the ladder amplitudes coincide with the ones of Eq. (S11) for the monochromatic case. Please notice that the same limit is reached with $\sigma_x \to 0$, but in this case XUV spectral bandwidth goes to infinity and Eq. (S39) is no longer valid.

S2.8 Comparison with SFA calculations

To test the result of Eq. (S58), we performed SFA calculations as described in Sec. S2.1 and compare the value of Λ_n^2 predicted by the model with the one directly extracted from the simulated spectrogram. To mimic the experimental conditions we simulated the single harmonic spectrogram (SHS) generated by harmonic 23 in Neon with an XUV time duration $\sigma_x = 11\,\text{fs}$ and an IR time duration, σ_r, that varies between T_{IR} and $22T_{IR}$ with $T_{IR} = 2.6685\,\text{fs}$ (i.e. corresponding to a wavelength of 800 nm). We repeated the calculations for different IR intensities

in the range between 10^8 and 10^{12} W/cm^2. An example of different SHSs at the experimental IR intensity of 5×10^{11} W/cm^2 and for transform limited (TL) IR and XUV pulses is reported in Fig. S10. As expected, the numerical simulations confirm that the strength of the SBs depends on the IR time duration. The quantity Λ_n^2 is extracted from the simulated spectrograms by inte-

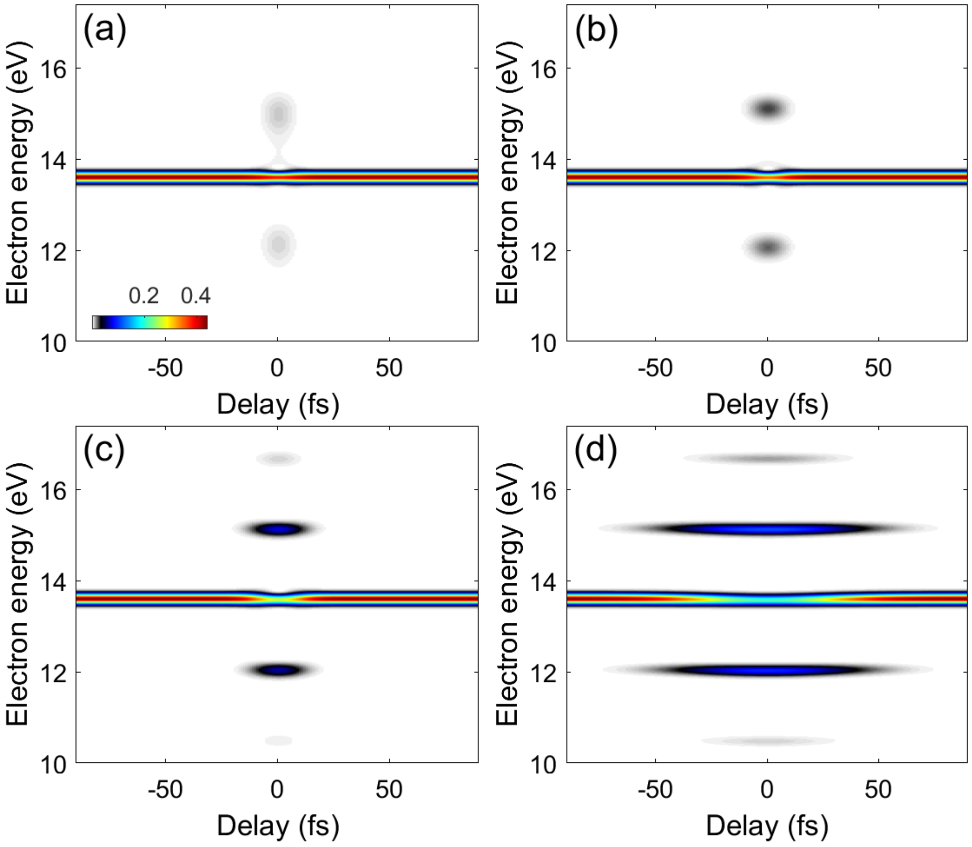

Figure S10: SHSs calculated for H23 in Neon with $\sigma_x = 11$ fs (TL). The IR average intensity is set to $I_{IR} = 5 \times 10^{11}$ W/cm^2 while $\sigma_{IR} = 1T_{IR}$ **(a)**, $2T_{IR}$ **(b)**, $4T_{IR}$ **(c)** and $22T_{IR}$ **(d)**.

grating the n-th SB signal around its nominal energy position in a 1.1-eV energy window and evaluating the maximum of the resulting delay-dependent curve. At low intensity, the maximum is located at $\tau = 0$ fs. As done for the experimental data, the extracted value is normalized by the area of the XUV spectrum, following what defined in Eq. (S58). Figure S11 reports a comparison between the amplitudes Λ_n^2 extracted from the SFA calculations and calculated with the

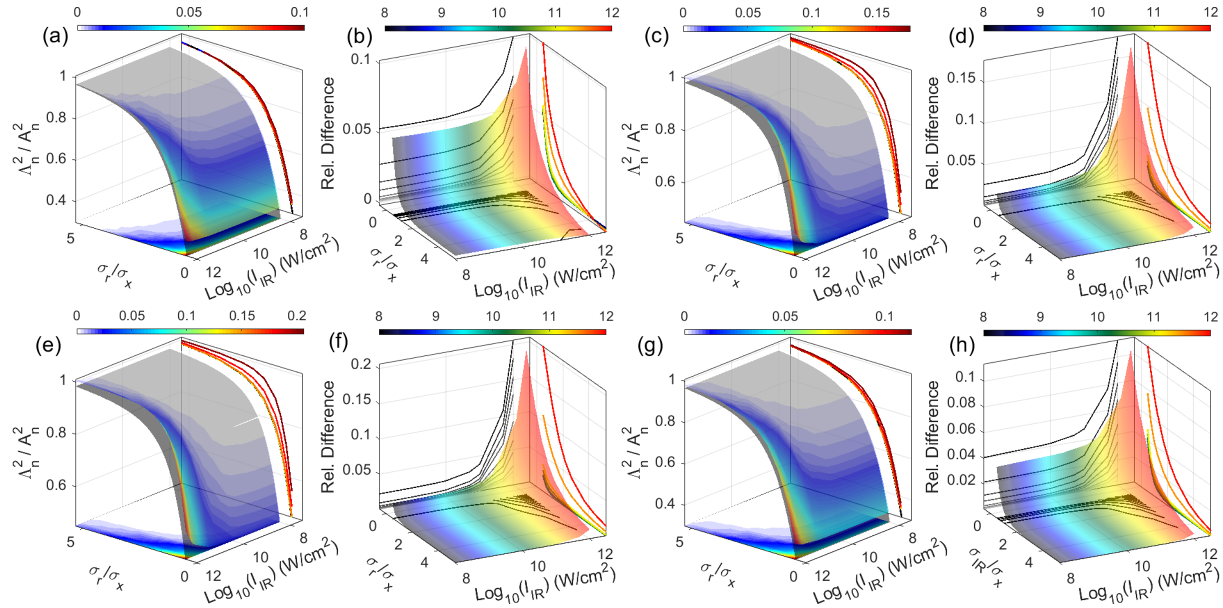

Figure S11: Comparison between the normalized SB amplitude Λ_n^2/A_n^2 calculated with SFA or with Eq. (S58) for σ_r/σ_x between 0.5 and 5.33 and $10^8 < I_{IR} < 10^{12}$ W/cm^2. In all the calculations $\sigma_x = 11$ fs while the IR duration is scanned between $2T_{IR}$ and $22T_{IR}$. Each couple of figures corresponds to a SB order. **(a)** and **(b)** are for $n = -2$, in **(c)** and **(d)** $n = -1$, in **(e)** and **(f)** $n = 1$ and **(g)** and **(h)** for $n = 2$. The first figure of each couple presents the Λ_n^2/A_n^2 obtained with Eq. (S58) (black surface) and with the SFA model (colored surface). The color scale indicates the difference between the two curves, Δ_n, depicted also by the contour plot at the bottom of the figure. The lines in the rear plane represent the Λ_n^2/A_n^2 at selected intensities as extracted from the SFA simulations. The second figure in the couple shows the corresponding relative difference defined as $(\Lambda_n^2 - A_n^2)/A_n^2$. The curves at the bottom show the contour lines of the represented surface while the lines in the rear vertical panels show the projections at fix intensities or durations. Both for the surface and for the lines in the right rear panel, false colors represent $\log_{10}(I_{IR})$.

analytical expression of Eq. (S58). In order to allow a direct comparison between the SB amplitudes obtained at different intensities, we decided to plot the quantity Λ_n^2/A_n^2 (Figs. S11(a), (c), (e), (g)). As it is possible to notice the SFA calculations (colored surfaces and curves) nicely agree with the theoretical model (black surface and curve) at the low intensity and long pulse limit. For short IR pulse durations or for IR intensities higher than 10^{11} W/cm^2 the SFA results are generally higher than what predicted by the analytical model. The larger error between SFA and theory is observed for the first SB ($n = 1$, Figs. S11(e) and (f)). Nevertheless, at the average IR intensity (5×10^{11} W/cm^2) and for the σ_r/σ_x range used in the experiment, the relative error, $(\Lambda_n^2 - A_n^2)/A_n^2$, stays below 8% (Fig. S11(f)). For the second SB ($n = 2$, Fig. S11(g)) the error stays below 5% (Fig. S11(h)). While all the above calculations have been performed with transform-limited pulses, the validity of Eq. (S58) has been tested against a finite GDD of both pulses. The degree of agreement found is comparable to what reported in Fig. S11 for TL pulses. If the actual time duration of the pulse is considered, the model predicts the correct SB strength behavior also for higher chirp orders, unless the spectral chirp is so severe to cause a clear deviation of the pulse temporal envelope from a Gaussian shape.

S2.9 Two-time approach

We here show how the approach used in Ref. (*9*) can be used to justify Eq. (S36). Let's start by introducing the function

$$V(t) = e^{i\frac{pE_0(t)}{\omega_0^2}\sin(\omega_0 t+\varphi)}. \tag{S60}$$

If the pulse envelope evolves on a slower time scale than the sinusoidal part, then we can introduce a second time t' which defines a new quantity

$$\bar{V}(t,t') = e^{i\frac{pE_0(t)}{\omega_0^2}\sin(\omega_0 t'+\varphi)}. \tag{S61}$$

With this, we can write the Fourier expansion of $V(t)$ where the amplitudes of the expansion are calculated by considering the periodicity of $\bar{V}(t,t')$ in t'. We get:

$$V(t) = \sum_m \tilde{V}_m(t) e^{im\omega_0 t} \tag{S62}$$

$$\tilde{V}_m(t) = \frac{1}{T_{\omega_0}} \int_0^{T_{\omega_0}} dt' \bar{V}(t,t') e^{-im\omega_0 t'} \tag{S63}$$

where indeed

$$V(t) = \sum_m \tilde{V}_m(t) e^{im\omega_0 t} = \sum_m \left(\frac{1}{T_{\omega_0}} \int_0^{T_{\omega_0}} dt' \bar{V}(t,t') e^{-im\omega_0 t'} \right) e^{im\omega_0 t} = \tag{S64}$$

$$= \frac{1}{T_{\omega_0}} \int_0^{T_{\omega_0}} dt' \bar{V}(t,t') \sum_m e^{-im\omega_0 (t'-t)} = \frac{1}{T_{\omega_0}} \int_0^{T_{\omega_0}} dt' \bar{V}(t,t') \delta(t'-t) = V(t). \tag{S65}$$

Using Eq. (S61) and Eq. (S63) we can now write

$$\tilde{V}_m(t) = \frac{\omega_0}{2\pi} \int_0^{\frac{2\pi}{\omega_0}} dt' e^{i\frac{pE_0(t)}{\omega_0^2}\sin(\omega_0 t' + \varphi)} e^{-im\omega_0 t'}, \tag{S66}$$

from which, using the Jacoby-Anger expansion we get

$$\tilde{V}_m(t) = \frac{\omega_0}{2\pi} \int_0^{\frac{2\pi}{\omega_0}} dt' \sum_{n=-\infty}^{\infty} J_n\left(\frac{pE_0(t)}{\omega_0^2}\right) e^{in(\omega_0 t' + \varphi)} e^{-im\omega_0 t'} = \tag{S67}$$

$$= \frac{\omega_0}{2\pi} \sum_{n=-\infty}^{\infty} J_n\left(\frac{pE_0(t)}{\omega_0^2}\right) e^{in\varphi} \int_0^{\frac{2\pi}{\omega_0}} dt' e^{i(n-m)\omega_0 t'}. \tag{S68}$$

The last integral in the above equation differs from zero only if $m = n$ where it equals the period. Therefore we get

$$\tilde{V}_m(t) = \frac{\omega_0}{2\pi} J_m\left(\frac{pE_0(t)}{\omega_0^2}\right) e^{im\varphi} \frac{2\pi}{\omega_0}. \tag{S69}$$

If now we insert this into Eq. (S62) we get:

$$V(t) = \sum_m \tilde{V}_m(t) e^{im\omega_0 t} = \sum_m J_m\left(\frac{pE_0(t)}{\omega_0^2}\right) e^{im\varphi} e^{im\omega_0 t} \tag{S70}$$

which says that in the SVEA we can approximate

$$e^{i\frac{pE_0(t)}{\omega_0^2}\sin(\omega_0 t+\varphi)} = \sum_{m=-\infty}^{\infty} J_m\left(\frac{pE_0(t)}{\omega_0^2}\right) e^{im(\omega_0 t+\varphi)} = \sum_{n=-\infty}^{\infty} J_n\left(-\frac{pE_0(t)}{\omega_0^2}\right) e^{-in(\omega_0 t+\varphi)} \tag{S71}$$

which is the dual of the Jacobi-Anger formula for monochromatic fields:

$$e^{i\frac{pE_0}{\omega_0^2}\sin(\omega_0 t+\varphi)} = \sum_{m=-\infty}^{\infty} J_m\left(\frac{pE_0}{\omega_0^2}\right) e^{im(\omega_0 t+\varphi)} = \sum_{n=-\infty}^{\infty} J_n\left(-\frac{pE_0}{\omega_0^2}\right) e^{-in(\omega_0 t+\varphi)}. \tag{S72}$$

Supplementary References

1. L. Poletto, P. Villoresi, F. Frassetto, F. Calegari, F. Ferrari, M. Lucchini, G. Sansone, and M. Nisoli, "Time-delay compensated monochromator for the spectral selection of extreme-ultraviolet high-order laser harmonics," *Review of Scientific Instruments*, vol. 80, p. 123109, dec 2009.

2. M. Lucchini, G. D. Lucarelli, M. Murari, A. Trabattoni, N. Fabris, F. Frassetto, S. De Silvestri, L. Poletto, and M. Nisoli, "Few-femtosecond extreme-ultraviolet pulses fully reconstructed by a ptychographic technique," *Optics Express*, vol. 26, p. 6771, mar 2018.

3. M. Nisoli, S. De Silvestri, and O. Svelto, "Generation of high energy 10 fs pulses by a new pulse compression technique," *Applied Physics Letters*, vol. 68, no. 20, pp. 2793–2795, 1996.

4. K. W. DeLong, R. Trebino, J. Hunter, and W. E. White, "Frequency-resolved optical gating with the use of second-harmonic generation," *Journal of the Optical Society of America B*, vol. 11, p. 2206, nov 1994.

5. M. Murari, G. D. Lucarelli, M. Lucchini, and M. Nisoli, "Robustness of the ePIE algorithm for the complete characterization of femtosecond, extreme ultra-violet pulses," *Optics Express*, vol. 28, p. 10210, mar 2020.

6. M. Kitzler, N. Milosevic, A. Scrinzi, F. Krausz, and T. Brabec, "Quantum Theory of Attosecond XUV Pulse Measurement by Laser Dressed Photoionization," *Physical Review Letters*, vol. 88, p. 173904, apr 2002.

7. D. M. Wolkow, "Über eine Klasse von Lösungen der Diracschen Gleichung," *Zeitschrift für Physik*, vol. 94, pp. 250–260, mar 1935.

8. L. B. Madsen, "Strong-field approximation in laser-assisted dynamics," *American Journal of Physics*, vol. 73, pp. 57–62, jan 2005.

9. L. Medišauskas, U. Saalmann, and J.-M. Rost, "Floquet hamiltonian approach for dynamics in short and intense laser pulses," *Journal of Physics B: Atomic, Molecular and Optical Physics*, vol. 52, p. 015602, dec 2018.

10. B. Moio, G. L. Dolso, G. Inzani, N. Di Palo, R. Borrego-Varillas, M. Nisoli, and M. Lucchini, "Time-frequency mapping of two-colour photoemission driven by harmonic radiation," *Journal of Physics B: Atomic, Molecular and Optical Physics*, vol. 54, p. 154003, aug 2021.
